# Observations on Certain of the Diseases of Young Children

**Published:** 1851-01

**Authors:** 


*Observations on certain of the Diseases of young Children.* By CHARLES D. MEIGS, M. D., Prof. of Midwifery and the diseases of women and children, in the Jefferson Medical College at Philadelphia, etc., etc., etc. Philadelphia: LEA & BLANCHARD. 1850. 8 Vol. pp. 214.

This work, as the author states in the preface, contains the substance of several lectures delivered in the Jefferson College, in October, 1849, during the term preliminary to the session of 1849-50. It is not designed as a systematic treatise, "seeing," as the author remarks, "that the place is already occupied with numerous valuable books, presenting a complete body of doctrines on children's diseases."

To assure the reader that he will find in this small treatise many valuable observations, is only to echo the anticipations of every practitioner who is conversant with the previous publications of the author. Having enjoyed a long and large experience, much of which, it is to be presumed, has related specially to diseases of women and children; and been for many years a distinguished teacher of medicine in its applications to these diseases, it would be strange, indeed, if his contributions to our science and art were to prove unacceptable or uninstructive. Moreover, it is gratuitous to say that there are few among those who adorn the medical profession of this country, whose writings and teachings evince greater learning, research, scholarship, and talent, than do the publications and lectures of Prof. Meigs. We admire the fervor of his style, and the high professional tone which he assumes and maintains.

But all the excellencies that have been mentioned, do not secure the mind against error, in the prosecution of scientific inquiries; and, indeed, some of the qualities which serve most to attract, and to enlist the sympathies of the hearer, or reader, are apt to mislead in the pursuit of truth. Enthusiasm, which, within just bounds, serves to stimulate exertion, and inspire zeal, by a natural and easy transition may pass into extravagance and credulity. The phenomena of science, even of the science of disease, present abundance of material for the exercise of a vivid imagination, often luring the hardy way-farer to turn aside from the tedious and rugged paths of observation, and induction, with the vain hope to anticipate results by a shorter route, and to enjoy fruits which, however pleasing to the eye, may prove to be but ashes to the taste.

In perusing the "observations on certain of the diseases of young children," we have found, with much that is interesting and useful, not a little that furnishes fair occasions for criticism. If the work has striking excellence, it has faults not less glaring. The latter will be as obvious to the intelligent medical reader, as the former. The private teacher, in putting the book into the hands of his pupil, will find it necessary to interpose a caution against receiving the peculiar views of the author, as established or accredited truth. The advise of most persons would probably be, to attend to the practical maxims and suggestions, but read with distrust the portions in which the author launches into the sea of speculation.

We had not designed, in this notice, to have directed attention to particular portions of the work, which, on the one hand, appear to us of special practical value, and, on the other hand, obnoxious to criticism. We will, however, cite a few passages in illustration of the justness of the remark just made, assuring the reader that while we do not, by any means, quote all those to which exceptions might be taken, we also leave untouched not a little that will commend itself to his approbation.

Speaking of *diagnosis*, in Chapter First, the author enforces, in his glowing style, the importance of understanding the natural language of disease, involved in the manifestations of diseased organs—a study highly important in the diseases of children, since we have not the advantage of the statements of the patient, which, as the author justly remarks, are not always an advantage, the organs, irrespective of intellect, often being more truthful than verbal descriptions of morbid sensations dictated by the consciousness of the sufferer. In exemplification of the precept just explained, he gives the following:—

"How do you do, to-day?" said I to a lady.

"I am very sick, indeed, doctor."

"How are you sick—where are you sick?"

"I have had a terrible chill, which made my teeth chatter together, followed by fever, violent headache, pains in the back and limbs, and unappeasable thirst; I am dreadfully ill.

"Have you pain in the abdomen?"

"No."

"Have you any pain in the thorax?"

"No."

"Pain in the great joints?"

"No."

"Have you not a lump in your breast?"

"No, I have not."

"Yes, you have."

"Indeed, I have not."

"No." said a witness, "I have examined it with the greatest care.

"When was it examined?"

"Just now."

"Will you let me examine it?"

"Yes."

"Well, then, does not that hurt you?" I touched the breast.

The answer was an outcry; she had a lump in her breast; she had a *weed* in her breast, and did not know it. Suppose she had been dumb and deaf—would there have been any bar to my diagnosis in her surdmutism?

A friend of mine had lodgings for the summer, twenty miles from the city; his daughter, two years old, was seized with a fit of unextinguishable crying; she screamed all the time that she was awake.

On the morning of the second day, as her distress continued, he became much alarmed, and resolved, accompanied by his lady, to bring his child to me. They arrived at their city residence, and sent for me by an urgent messenger. I heard the child's voice from the third story, while I was in the lower hall. My friend began to explain the nature of the case, and to set forth all his alarm for his dear daughter. I stopped him, saying, "I hear her crying now, do I not?"

"Yes, that is her voice; she is in the greatest distress."

"She is crying with the ear-ache," said I.

"Ear-ache? How do you know that, doctor?"

"Come with me to the nursery, and I will prove it to you," said I; "I know the voice of the ear-ache."

When we came into the room, the child, surprised by my presence, ceased for a moment to scream. "Now," said I, "see if I don't prove to you that she has an ear-ache." I approached her, and putting the palp of my thumb on the right meatus auditorius, I suddenly pressed the cartilaginous tube inwards upon the ear; the child merely looked surprised at my rudeness; she did not scream. "It is not the right ear," said I. I next repeated the same movement as to the left ear, and she screamed as if she would go into convulsions. "There," said I, "I have hurt her ear for you, by a slight touch, and she cries with the same voice that I heard when I was down stairs; I knew that it was the ear-ache then, and I as sure of it now, this touch is the diagnostical test." The cry was a test."

These anecdotes are sufficiently in point as illustrations of the diagnostic prominence of two isolated symptoms; but we must take serious exceptions to the inferences which the young student would be likely to draw from such relations.

Does Prof. M. really mean to inculcate the notion, that a patient having experienced a chill, without pain in the abdomen, thorax, or great joints, must necessarially have a *weed* in her breast? Does he mean to teach that his students are to do, in similar cases, as he did in that case, pronounce upon the diagnosis so promptly, and positively, and with no other data? Or, is he willing to be understood that an ear-ache is to be diagnosed from the cry of a child heard from the third story? If he is accustomed to draw conclusions in that way, as to the seat and nature of disease, he should, in fairness, have given other instances in which he failed to *guess* rightly, for, after all, it is guessing, shrewdly, intelligently mayhap, but still guessing.

The advice, by the same author, given in another work, as it seems to us' conveys a safer and better maxim. "I advise the student early to come to the resolution of being cautious in giving his diagnosis and prognosis of these doubtful cases of labor. I know that there belongs to professional men a disposition to *pronounce* at once. This, perhaps, arises from a false pride, which prompts them to seem to know all things at a glance, or by mere intuition."—Meigs' Treatise on Obstetrics, page 262.

In the chapter on Cyanosis neonatorum, after assuming that the nervous mass is the creature, the *Ens*, in all living beings, the rest of the organism being "vile, common, and of less account"—which possibly might be entertained as a philosophical conceit, or a poetical license, he proceeds as follows:—

"I shall not encumber these pages with quotations from the authorities to fortify the assertion that the nervous mass is the essential Ens. The asseverations of a thousand philosophers would not make more or less true a proposition which commends itself to the mind, acting upon its own perception and judgment of a dogma declared to be true. Such a truth is not proved by evidence, nor established by any method of induction. It is a truth of reason—it is a truth of consciousness—it is in the same category with the cognition of our personal identity."

We leave with the reader, without comment, the idea that a physiological proposition is not to be "proved by evidence, nor established by any method of induction," but is to be classed among the intuitive truths, and placed in the same category with the belief in personal identity! This is transcendentalism *par excellence.*

In the same chapter the reader will observe a formal argumentation of the point that it is not blood that develops the nervous force inherent in the cerebro-spinal axis, but the oxygen that the blood contains. The author contends that it is not a "mere fancy in Oken to say that 'the artery is an air-tube.'" He is not content with the position that oxygenated blood is requisite to produce the nerve force, but insists that it is oxygen and not blood! This metaphysical quibble, it would seem, in the author's estimation, involves a doctrine of great importance to the medical student.

The author's attachment to Oken, by the way, he admits, with a candor which is certainly as creditable as remarkable, is so great as to prevail over the exercise of his own judgment. He says:—

"For my own part, without feeling in general bound to adopt whatever explanations or rationales of life may be presented to me by men we call authorities, I freely admit that there is in me a tendency to surrender my judgment to the dicta of Lorenz Oken, notwithstanding the salutary declaration of St. Augustin, "*Quod scimus debemus rationi; quod credimus, auctoritati;*" expressions that ought to serve as a motto and general declaration of independence for all persons devoted to scientific pursuits. But of such authorities as Oken, it might almost be correct to say, in believing after him, *quod credimus scimus.*"

He regards, in fact, the opinions of his private author as "the basis of physiological truth." He adds following the passage just quoted:—

"Taking the foregoing declarations and statements of those distinguished physio-philosophers as a basis of physiological truth, the question issues from it as to what are the nature and extent of the force with which nervous mass is endowed."

Arguing in behalf of the insufficiency of evidence of the contagiousness of Scarlatina, we find the following remarks:

"I do not conceive that we are bound to believe, because two or more persons in one company or household are seized with scarlatina within a certain time after communication held with a patient laboring under it, they acquired it from the said person—not even if the same sort of circumstance should be observed a thousand consecutive times.

"Although it may be true that the cause of scarlet fever is a miasm, exhalation, gas, or substance, which is extricated from the bodies of the sick—yet if it is true, no one has hitherto proved it to be so. The most that can be said of it, therefore, is that it is in general supposed to be contagious."

If a thousand consecutive instances of the development of a disease, within a certain time, after communication held with a peres on laboring under it, be not evidence of contagion, we should like to know how the personal communicability of any disease is to be established irrespective of inoculation.

That the author refuses to recognise this sort of evidence, is only susceptible of explanation, on the ground that he is predetermined, in spite of any evidence, to deny the contageousness of scarlatina. This, indeed, he distinctly confesses, with admirable frankness, in the following passage:—

"My own mind has, for many years past, been so fully made up on this subject, that I do not suppose I shall ever change it so as to believe that scarlatina is propagated by contagion. I think that I have come to this conclusion after very careful consideration of the subject, with which a medical life, so long and active as mine has been, could not but have familiarized me."

We will content ourselves with one quotation more, which will serve to show the logical process by which the author arrives at certain notions in pathology. As a specimen of a *facile* method of reasoning, the following extract is certainly rich. The author dismisses, it will be perceived, the explanation which first suggests itself, because it is not agreeable to him to admit it. He is "hardly willing to attribute," etc. It would seem that truths of natural science are to be determined by volition, as well as intuitive *belief*! The next explanation is difficult to be entertained; the third conjecture is therefore probable; and, after all this "it remains to suppose!" but to the quotation—the author is speaking of the pathology of swelled liver in the jaundice of infants:—

"I am unacquainted with the precise principles of the pathology of this condition; I am hardly willing to attribute it to faults of a simple excessive sanguine determination to the capillary termini of the hepatic artery; and it is equally difficult to assign as a cause for it, any affection of the hepatic veins; it is probably, therefore, a fault essentially resident in the capillary termination of the hepatic portæ. It only remains to suppose that the sanguine engorgement is to be referred to a fault in the condition of the digestive circulations which issue from the aorta—that is, by the cœliac and the two mesenterics, hence, the farther inference that the trouble in the hepatic functions is pathologically connected with some faulty action of the capillary ramifications of the three digestive arteries above named, or of the venous capillaries of their second expansions in the substance of the biliary organ."

As already remarked, we have cited the foregoing extracts by way of illustration of the correctness of the opinion, previously expressed, that the work is not to be recommended to medical students in unqualified terms. Books, like individuals, have always their faults. Sustaining the comparison, we would say, that in this instance, the eccentricities, which are so numerous and striking, may perhaps be overlooked in consequence of the good qualities which make it acceptable.

For sale by Phinney & Co.

